# The Difference of Volatile Compounds in Female and Male Buds of *Trichosanthes anguina* L. Based on HS-SPME-GC-MS and Multivariate Statistical Analysis

**DOI:** 10.3390/molecules27207021

**Published:** 2022-10-18

**Authors:** Pingping Song, Bo Xu, Zhenying Liu, Yunxia Cheng, Zhimao Chao

**Affiliations:** Institute of Chinese Materia Medica, China Academy of Chinese Medical Sciences, Beijing 100700, China

**Keywords:** *Trichosanthes anguina*, HS-SPME-GC-MS, monoecious and diclinous plant, bud, volatile compound, OPLS-DA

## Abstract

*Trichosanthes anguina* L. (family Cucurbitaceae) is a monoecious and diclinous plant that can be consumed as a vegetable and has anti-inflammatory and antioxidant effects. The chemical composition and content of volatile compounds in female and male buds of *T*. *anguina* were explored by headspace solid-phase microextraction-gas chromatography-mass spectrometry (HS-SPME-GC-MS) technology combined with multivariate statistical analysis. The results showed that the content of the volatile compounds was different between female and male buds. 2,2,6-trimethyl-6-vinyltetrahydro-2H-pyran-3-ol and 2,2,6-trimethyl-6-vinyldihydro-2H-pyran-3(4H)-one were the main volatile compounds in both female and male buds. Based on the multivariate statistical analysis of orthogonal projections to latent structures discriminant analysis (OPLS-DA) and *t*-test, the content of seven compounds was significantly different between female and male buds. The content of three compounds in male buds was higher than that in female, i.e., (*E*)-4,8-dimethyl-1,3,7-nonatriene, 1,5,9,9-tetramethyl-1,4,7-cycloundecatriene, and (*E*)-caryophyllene. Conversely, the content of (*Z*)-4-hexen-1-ol, (*Z*)-3-hexenyl benzoate, (*Z*)-3-hexenyl salicylate, and 2-hexen-1-ol in female buds was higher than that in male buds. This is the first report on the difference in the volatile compounds between female and male buds of *T*. *anguina*, which enriches the basic research on the monoecious and diclinous plant and provides a reference for the study of plant sex differentiation.

## 1. Introduction

*Trichosanthes anguina* L. (family Cucurbitaceae), an annual climbing herb, is known as snake gourd or snake bean for its slender (up to 200 cm in length and 3 cm in diameter), twisted, and snake-like shape fruit. It is originated in India and Malaysia and commonly cultivated in tropical and subtropical areas and northern China [1]. Its tender fruit is a popular vegetable, which contains proteins, carbohydrates, cellulose, fat, and a variety of minerals [2]. The fruit, seed, and root of *T*. *anguina* can be used in traditional Chinese medicine for some effects in clearing heat and generating fluid, moisturizing the lung, eliminating dampness, and destroying parasites [3]. It has anti-inflammatory and antioxidant activities and can be used for the treatment of malaria and bronchitis [4]. In addition, two proteins of TR3 from the root and of TS3 from the seed have cytotoxic activity on cancer cell lines [5].

The seed of *T*. *anguina* contains 3% free sugar, 7% starch, 20% protein, and 43% fat oil, which consists of punicic acid, palmitic acid, stearic acid, oleic acid, and linoleic acid [6,7]. The fruit contains chlorogenic acid, isochlorogenic acid, *p*-coumaric acid, vanillic acid, ferulic acid, protocatechuic acid, caffeic acid, salicylic acid, phloretic acid, 3-indole acetic acid, phloroglucinol, quercetin, calcium, iron, phosphorus, folic acid, carotene, and vitamins B1, B2, and C [8,9]. The leaf contains kaempferol-3-*O*-*β*-galactoside and kaempferol-3-*O*-*β*-sophoroside [10].

Most flowering plants are bisexual plants whose flowers have both pistils and stamens. In order to avoid the decrease in the survival adaptability of offspring caused by self-inbreeding or self-mating [11], some plants evolved to unisexual; that is, there are only pistils or stamens in each flower, called female flower or male flower. The plant of which female and male flowers grow in different plants is called a dioecious plant, but in the same plant is known as a monoecious and diclinous plant. There are many differences in apparent structure, physiological function, and chemical components between female and male flowers of dioecious plants, such as *Populus tomentosa* (family Salicaceae) [12], *Herpetospermum pedunculosum* (family Cucurbitaceas), and *T. kirilowii* [13,14]. For monoecious and diclinous plants, the difference between female and male flowers also exists; for example, the chemical compounds from flowers of *Cucurbita moschata* (family Cucurbitaceas) [15] and the flower morphology of *Croton sarcopetalus* (family Euphorbiaceae) [16]. However, there are few comparative reports on the difference between female and male flowers of some monoecious and diclinous plants up to now.

**Figure 1:**
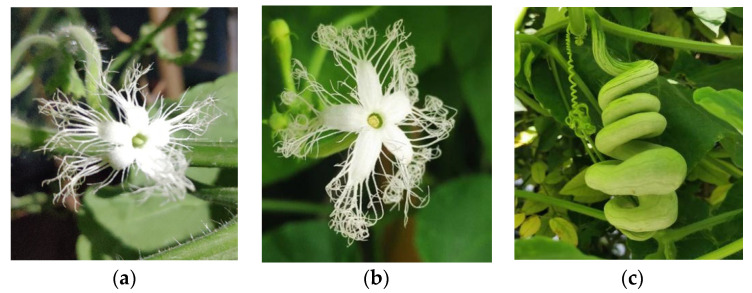
The female flower (**a**), male flower (**b**), and tender fruit (**c**) of *T*. *anguina*.

The plant of *T*. *anguina* is one of the monoecious and diclinous plants. Its calyxes are green, five-lobed, and villous. Both female and male flowers bloom at night, but the blooming time of females is later than that of male flowers. Their corollas are pale yellowish green at the initial opening and turn white at full opening, five-lobed, and with branched and curly terminals. Female flowers are solitary and male flowers are raceme. Female flowers are 5–6 cm long and located at the top of juvenile fruit with a green pistil, two-lobed stigma, and inferior ovary. Male flowers are 3–5 cm long with three stamens and connate anthers. The female and male flowers and tender fruit are shown in Figure 1. The chemical constituents of female and male buds of *T. anguina* were studied, and the difference in sex was compared to make up for the deficiency of sex study of the monoecious and diclinous plants.

Solid-phase microextraction (SPME) is a sample treatment method invented by Pawliszyn and Arthur in the 1990s [17], which needs no solvent to extract the sample components. The SPME fiber is used to extract samples directly whose weight is usually less than 5 mg [18]. Headspace (HS) technology combined with SPME is a convenient extraction method. The sample is directly placed in a sealed headspace bottle, and an SPME fiber is put into the headspace bottle and located above the sample. During the heating and extraction process, volatile substances diffuse from the sample to the SPME fiber and are enriched in the fiber. This extraction method is so quick, less than one hour, and does not destroy the sample or require a liquid solvent. HS-SPME is often combined with gas chromatography-mass spectrometry (GC-MS) for widespread use in some volatile substances analysis [19,20].

The material of SPME fiber coating is crucial to the extraction, which determines the fiber’s affinity to compounds in the sample. Since the introduction of SPME, many coating materials have been employed in the analysis. Polydimethylsiloxane (PDMS) fiber could be used to determine the content of acrylamide from coffee beans [21]. Polyacrylate (PA) fiber was suitable for the quantification of sesquiterpenes in *Zingiber zerumbet* L. (family Zingiberaceae) volatiles [22]. Polydimethylsiloxane/divinylbenzene (PDMS/DVB) could be applied for the determination of the esters of carboxylic acids in insect lipids [23]. Carboxen/polydimethylsiloxane (CAR/PDMS) fiber was used for the analysis of volatile compounds from European ciders [24]. Divinylbenzene/carboxen/polydimethylsiloxane (DVB/CAR/PDMS) fiber was able to extract more chemical diverse volatile compounds, including ketones, aldehydes, alcohols, terpenoids, and others from *Medicago sativa* L. (family Leguminosae) compared with PDMS and CAR/PDMS fibers [25] and to analyze a variety of plants and their oils such as sweet potato, tomato, and olive oils [26,27,28]. In this study, DVB/CAR/PDMS fiber was selected due to its better ability to provide a more comprehensive chemical profile of plants and its great extraction effect in the pre-experiment.

The buds, just before blooming, were picked up as experimental samples for the detection of volatile compounds in order to avoid any contamination of external substances. The HS-SPME-GC-MS method was used to detect and identify the volatile compounds from the female and male buds of *T*. *anguina*. The multivariate statistical analysis of OPLS-DA and *t*-test were carried out to screen out the differential compounds between female and male buds for revealing the difference in the chemical composition of genders of monoecious and diclinous plants.

## 2. Results

### 2.1. Volatile Compounds of GC-MS Analysis

**Figure 2:**
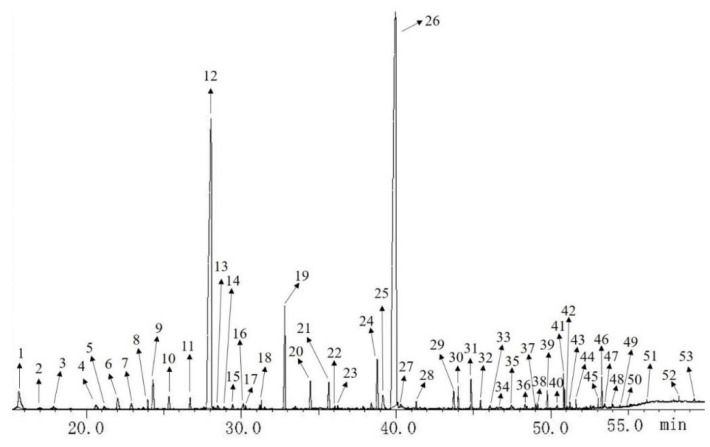
GC-MS total ion chromatogram of volatile compounds of sample f2.

**Figure 3:**
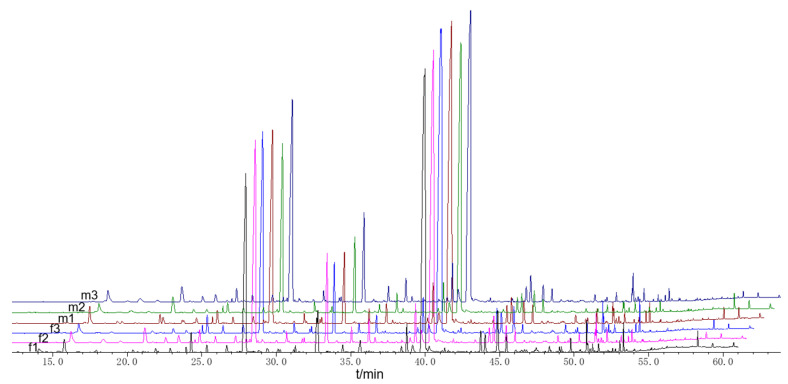
GC-MS total ion chromatograms of volatile compounds from female and male buds of *T. anguina*.

Both female and male fresh buds of *T. anguina* just before blooming were individually picked up at night. The samples were weighed as f1 0.1063 g, f2 0.1057 g, f3 0.0975 g, m1 0.0842 g, m2 0.0826 g, and m3 0.0849 g. The volatile compounds were analyzed by HS-SPME-GC-MS successfully. The total ion chromatography is shown in Figure 2. A total of 53 compounds were identified on the base of mass spectrum and retention index (RI). The relative content of these volatile compounds was calculated with peak area normalization and shown in Table 1. The total ion chromatograms of volatile compounds for three female and three male bud samples are shown in Figure 3.

2,2,6-Trimethyl-6-vinyltetrahydro-2H-pyran-3-ol (no. 26) (female 38.71–42.71% and male 39.62–42.64%) and 2,2,6-trimethyl-6-vinyldihydro-2H-pyran-3(4H)-one (no. 12) (female 27.44–35.82% and male 28.34–32.95%) were two compounds with the highest content of *T. anguina* buds.

The volatile components of female and male buds of *T. anguina* included alcohols, ketones, aromatic esters, non-aromatic esters, monoterpenes, sesquiterpenes, diterpenes, alkenes, oximes, heterocycles, alkanes, and acids. The detailed results are shown in Table 2. Among them, the type of heterocycles was the highest content component, with a total relative content of 69.75–75.49% for female and of 69.58–73.39% for male buds. The total content of alcohols was the second highest, with 9.48–12.36% for females and 7.59–8.89% for male buds. Interestingly, there were seven sesquiterpenes whose content in male buds (average 3.56%) was significantly higher than that in female buds (average 2.66%) with *p* < 0.05 in the *t*-test. In addition, the content of four alkenes in male buds (average 5.34%) was significantly higher than that in female buds (average 2.25%), with *p* < 0.05 in the *t*-test. These results showed that there was a significant difference between female and male buds in terms of the content of sesquiterpenes and alkenes.

### 2.2. Multivariate Statistical Analysis

**Figure 4:**
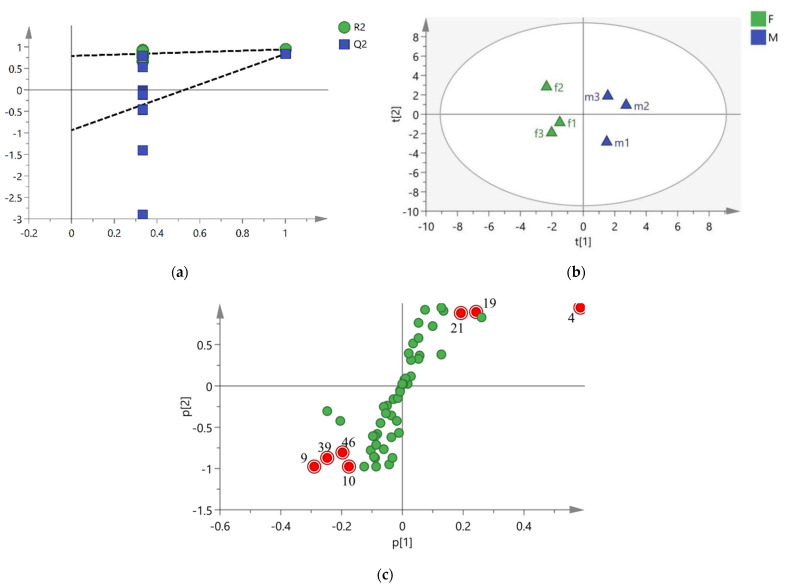
OPLS−DA arranges the verification diagram (**a**), scores (R^2^ = 0.944; Q^2^ = 0.838) (**b**), and S−plot (**c**) between female and male buds of *T. anguina*. The numbers of the S−plot in Figure (**c**) were consistent with the no. in Table 1.

An OPLS-DA model of multivariate statistical analysis was carried out in order to further explore the difference in volatile compounds between female and male buds. This model was verified by 200 times permutation tests. Figure 4a showed that the R^2^ and Q^2^ values generated by any random arrangement on the left end were smaller than those on the right end, the slope of the regression line was large, and the lower regression line intersected the negative half-axis of the *Y*-axis, indicating that the model was not overfitting and could be used to find differential compounds. The OPLS-DA score diagram (Figure 4b) showed that the points of f1−f3 of female samples and m1−m3 of male samples were separated along the t1 axis. The value of R^2^ was 0.944, and that of Q^2^ was 0.838, both of which were greater than 0.5, indicating that the model had suitable interpretation and prediction ability.

Seven compounds were screened out as differential compounds between female and male buds of *T. anguina*, whose VIP values were greater than 1 (Table 3), dots were far away from the origin in the S-plot (Figure 4c), and *p* values of *t*-test were less than 0.05. The content of three compounds in females was significantly lower than that in male buds, i.e., (*E*)-4,8-dimethyl-1,3,7-nonatriene (no. 4), 1,5,9,9-tetramethyl-1,4,7-cycloundecatriene (no. 21), and (*E*)-caryophyllene (no. 19). The content of (*Z*)-4-hexen-1-ol (no. 9), (*Z*)-3-hexenyl benzoate (no. 39), (*Z*)-3-hexenyl salicylate (no. 46), and 2-hexen-1-ol (no. 10) in female was higher than that in male buds. These seven differential compounds were marked in red in the S-plot (Figure 4c).

### 2.3. Heat Map

**Figure 5:**
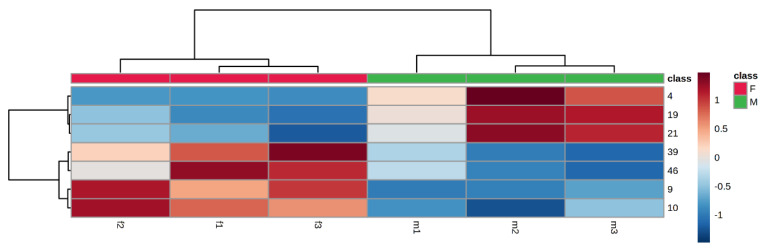
The heatmap of the differential compounds in female and male buds of *T. anguina*. The numbers of the heat map were consistent with the no. in Table 1.

The content distribution of seven differential compounds in the female and male buds of *T. anguina* was visualized in the form of a heat map. Horizontal columns represented different samples (f1–3 and m1–3), and vertical columns represented different compounds (no. 4, 19, 21, 39, 46, 9, and 10). If the color of the block was red, the deeper the red was, the higher the content of the compound in the sample was; if the color was blue, the deeper the blue was, the lower the content of the compound was. As shown in Figure 5, six samples were clearly separated into two groups, i.e., all three female samples were classified into a group on the left, and three male samples were classified into a group on the right. Therefore, the different content levels of seven differential compounds in the female and male buds could be intuitively and clearly observed in Figure 5.

## 3. Discussion

As a monoecious and diclinous plant, the structure and function of female and male buds and flowers of *T. anguina* are quite different. The buds just before opening were collected for our experiment, which confirmed some differences in the volatile compounds between female and male buds. The results must be more accurate and reliable than those of opening flowers, which can avoid the loss of bud volatile compounds and the pollution of external substances [13]. This is the first report to study the volatile compounds from the buds and to find the differential compounds between female and male buds of *T. anguina*.

Diisobutyl phthalate (no. 51) is usually used as a plasticizer, which is often detected in soil, water, and air, besides in plastics. It is a detection index component of environmental pollution [54]. In this experiment, the fresh buds were treated by HS-SPME without any solvent extraction or plastics exposure. Therefore, it was suspected to be derived from the soil during growth or from the air.

(*E*)-4,8-Dimethyl-1,3,7-nonatriene (DMNT) is one of the floral compounds of some night-flowering plants [55]. DMNT can attract insects to pollinate [56] and has the function of attracting natural enemies of pests to avoid pest invasion [57]. The average content of DMNT in males (3.48%) was 6.14 times that in female buds (0.57%), whose content gap between genders was the largest. The results pointed out that high DMNT content in male buds was more conducive to attracting insects to pollinate and improving the reproductive capacity of *T. anguina*.

(*E*)-Caryophyllene, a bicyclic sesquiterpene, has many pharmacological effects such as anti-inflammation, antidepression, and anti-convulsion [58]. Its average content in male (2.37%) was higher than that in female buds (1.81%), which is a significant difference with *p* < 0.05 in the *t*-test between female and male buds. The result indicated that the male buds should have stronger pharmacological activities than the female buds of *T. anguina*.

1,5,9,9-Tetramethyl-1,4,7-cycloundecatriene was detected, whose average content in male (1.42%) was higher than that in female (1.08%) buds and had a significant difference (*p* < 0.05 in *t*-test) in genders. It was often found in volatile components from some plants, such as *Zanthoxylum dissitum* (family Rutaceae) and *Artemisia dracunculus* (family Compositae) [39,59].

(*Z*)-3-Hexenyl benzoate and (*Z*)-3-hexenyl salicylate are two (*Z*)-3-hexenol esters of aromatic acids. The average content of the former was 1.26% in females and 0.64% in males, and of the latter was 0.92% in female and 0.47% in male buds. Both of their content in females was about two times that in male buds. (*Z*)-3-Hexenyl benzoate showed specific binding to odorant-binding proteins of *Halyomorpha halys* and *Plautia stali*, which was similar to alarm pheromones of both two pests with the function of repelling [60]. The higher content of (*Z*)-3-hexenylbenzoate in female buds indicated that the compound might help female flowers avoid pests and protect female flowers’ pollination and development. (*Z*)-3-Hexenyl salicylate is a useful compound for people, often used as fragrance ingredients for fine fragrances, shampoos, toilet soaps, and household cleaners [61].

2,2,6-Trimethyl-6-vinyltetrahydro-2H-pyran-3-ol was the most abundant compound of all the volatile compounds from *T. anguina* buds, whose content was 38.71−42.71% and 39.62−42.64% in female and male buds, respectively. It was also the main aroma compound from pu-erh teas, a popular fermented tea that originated in Yunnan [33]. The content of 2,2,6-Trimethyl-6-vinyldihydro-2H-pyran-3(4H)-one was the second highest volatile compound in both buds of *T. anguina*. It also had a high content in the volatile compounds of *Camellia sasanqua* ‘Dongxing’ flowers (family Theaceae) [62].

The chemical components of some dioecious plants were reported to be different between females and males. The essential oils of the female and male aerial parts of *Baccharis tridentata* Vahl. (family Asteraceae) were explored with GC-MS analysis. *α*-Pinene was the main compound in the essential oil of both genders, which was presented at higher content in males (1173 ± 60 μg/L) than that in females (794 ± 40 μg/L). Conversely, the concentrations of *α*-phellandrene, *α*-terpinene, and *trans*-verbenol in female essential oil were higher than those in males [42]. The chemical components from female and male flowers of *Schisandra chinensis* (family Magnoliaceae) were differentiated with GC-MS analysis. The results showed that 16 compounds were found only in female flowers (including *α*-farnesene, *α*-pinene, and 3-carene), and 19 compounds (including *p*-xylene, 3-pyridinecarboxaldehyde, and 1,2-epoxydodecane) were detected only in male flowers. A number of compounds detected both in female and male flowers were quantitatively different; for example, the content of *β*-pinene in females was 6.36 times that in male flowers (0.70 ± 0.06% and 0.11 ± 0.01%, respectively) [51].

The plant *T. kirilowii*, a different species of the same genus of *T. anguina*, was a dioecious plant. The highest content component of *T. kirilowii* flowers was alcohol, the same as *T. anguina* buds. Some same compounds of linalool, benzyl alcohol, and (*E*)-linalool oxide were found both in *T. anguina* and *T. kirilowii*. However, the highest content compound in females was linalool, but in male flowers of *T. kirilowii* was benzyl alcohol. Some compounds, such as *β*-myrcene and *α*-ocimene, were only detected in females, and benzaldehyde was only detected in male flowers of *T. kirilowii* [14]. The difference between female and male flowers of *T. kirilowii* was larger than that between female and male buds of *T. anguina*. In other words, the difference in volatile compounds between the two genders from the dioecious plant was larger than that from the monoecious and diclinous plants. Dioecious plants have more advanced evolution than monoecious and diclinous plants in plant sexology [63]. From the perspective of the flowers and buds of sexual organs, the higher inequality was found based on their volatile compounds.

## 4. Materials and Methods

### 4.1. Apparatus and Materials

A Shimadzu GC-MS-QP 2010 plus gas chromatography-mass spectrometer and a Swiss CTC Combi-xt PAL three-in-one multifunctional automatic sampler were purchased from Shimadzu (Tokyo, Japan). A polyethylene glycol capillary chromatographic column INNOWAX (30 m × 0.25 mm, 0.25 µm) was purchased from Shanghai Troody Analysis Instrument Co., Ltd. (Shanghai, China). The SPME fiber 50/30 µm carboxen/polydimethylsiloxane/divinylbenzene (CAR/PDMS/DVB) was purchased from Supelco (Bellefonte, PA, USA). An ME 204/02 electronic balance was purchased from Mettler Toledo Instruments Co., Ltd. (Shanghai, China). An *n*-alkanes standard (C_11_–C_32_) was purchased from Sigma-Aldrich (St. Louis, MO, USA).

### 4.2. Sample Collection

The plant of *T. anguina* was planted in the courtyard of 16 Dongzhimen South Street, Dongcheng District, Beijing (39°56′18.45′′ N, 116°25′41.06′′ E, and 48 m altitude). On 6th August, just before their buds bloomed, male buds were picked from pedicels at 8 p.m., and female buds were taken from the top of their juvenile fruits at 9 p.m. The original plant was identified as *Trichosanthes anguina* L. (family Cucurbitaceae) by Prof. Zhimao Chao (Institute of Chinese Materia Medica, China Academy of Chinese Medical Sciences) according to the description in Flora of China (Editorial Board of Flora of China, 1984). The voucher specimens (TAF 1–3 and TAM 1–3) were deposited at the 1022 laboratory of the Institute of Chinese Materia Medica, China Academy of Chinese Medical Sciences, Beijing, China.

### 4.3. Sample Preparation

The fresh bud samples were collected and placed in 15 mL glass headspace bottles. Each headspace bottle was placed in one of the bud samples, tightly covered, weighed, and subtracted from the bottle’s body weight to obtain the sample weight. Moreover, the material of the headspace bottle gasket was polytetrafluoroethylene (PTFE). The 50/30 µm CAR/PDMS/DVB SPME fiber was aged at 260 °C for 30 min, extended through the needle, exposed into the headspace bottle to adsorb volatile compounds at 50 °C for 30 min, and immediately injected into the gas chromatography injection port at 250 °C for 3 min to desorb volatile compounds.

### 4.4. Chromatographic Conditions

The volatile compounds of the samples were analyzed by the GC-MS method. A GC-MS-QP 2010 plus gas chromatography-mass spectrometer was used coupled to polyethylene glycol capillary chromatographic column Agilent HP-INNOWAX (30 m × 0.25 mm, 0.25 µm). The splitless injection mode was used. The carrier gas was high-purity helium, which was used at a constant flow rate of 1.01 mL·min^−1^. The temperature of the injection port was set at 250 °C. The heating program was as follows: the initial temperature was 40 °C maintained for 8 min, raised to 160 °C at a rate of 3 °C·min^−1^, and subsequently raised to 240 °C at a rate of 10 °C·min^−1^ and held for 5 min.

### 4.5. MS Conditions

The electron ionization (EI) source was used and operated at 70 eV. The ion source temperature was 200 °C. The interface temperature was 220 °C. Moreover, the scanning range was *m*/*z* 29–350.

### 4.6. Data Processing

The volatile compounds from samples were identified with the mass spectrum and RI. The mass spectra obtained from the GC-MS experiments were compared with the National Institute of Standards and Technology (NIST) 14 spectrum library. The RIs were calculated according to the peak retention time of these volatile compounds and of the series of *n*-alkanes (C_11_–C_32_) under the same temperament condition and were compared with the values in the previous reports. The peak area normalization was carried out for semi-quantitative analysis and for comparison of the difference of the volatile compounds between female and male samples.

### 4.7. Statistical Analysis

OPLS-DA is a supervised discriminant analysis that can effectively distinguish the difference between groups. The experimental data were imported into SIMCA-P software (version 14.1, Umetrics, Malmö, Sweden) to establish an OPLS-DA model to distinguish the female and male buds. The experimental data were also imported into SPSS (version 19.0, IBM, America), analyzed with a *t*-test, and then combined with OPLS-DA results to identify the significantly different compounds of the volatile components from female and male buds of *T. anguina*.

### 4.8. Heat Map

After data were imported into the Metabo Analyst 5.0 website, the heat map was generated to visualize the distribution of different compounds in different samples so that readers could intuitively observe the content gap of these compounds in female and male samples.

## 5. Conclusions

In this study, HS-SPME-GC-MS combined with multivariate statistical analysis was used to explore some differences in volatile compounds of *T. anguina* buds, and it was found that there was a significant difference between female and male buds. A total of 53 volatile compounds were identified by GC-MS. There were the same volatile compounds from female and male buds, but their content was different. Based on multivariate statistical analysis, seven different compounds were screened out. Among them, the content of (*E*)-4,8-dimethyl-1,3,7-nonatriene, (*E*)-caryophyllene, and 1,5,9,9-tetramethyl-1,4,7-cycloundecatriene from male was higher than that from female buds, and the content of (*Z*)-4-hexen-1-ol, (*Z*)-3-hexenyl benzoate, (*Z*)-3-hexenyl salicylate, and 2-hexen-1-ol from male was lower than that from female buds. Further comparison between the monoecious and diclinous plant of *T. anguina* and the dioecious plant of *T. kirilowii*, an opinion suggested that the difference of volatile compounds between female and male buds of the monoecious and diclinous plant was smaller than that of the dioecious plant. This opinion was consistent with the evolutionary view of plant sex [63].

This is the first report that the difference in volatile compounds between female and male buds of *T. anguina* was analyzed. Furthermore, a comparison between monoecious and diclinous plants and dioecious plants was first carried out.

## Figures and Tables

**Table 1:** Volatile compounds of GC-MS analysis of female and male *T. anguina* buds.

No.	RI	Compound	MF	Fragment (*m*/*z*)	CAS	Relative Content/%						Reports
						Female			Male			
						f1	f2	f3	m1	m2	m3	
1	1214	3-Methyl-1-butanol	C_5_H_12_O	87/70/57/55	123-51-3	2.00	2.67	2.29	2.58	2.41	2.30	-
2	1239	(*E*)-3,7-Dimethyl- 1,3,6-octatriene	C_10_H_16_	136/121/93/77	3779-61-1	0.21	0.18	0.12	0.09	0.13	0.19	Jasmine tea [29]
3	1256	1-Chloro-3-methylbutane	C_5_H_11_Cl	106/91/42/27	107-84-6	0.23	0.30	0.32	0.21	0.19	0.23	-
4	1311	(*E*)-4,8-Dimethyl-1,3,7- nonatriene	C_11_H_18_	150/135/69/53	19945-61-0	0.58	0.62	0.50	2.24	4.63	3.57	*Grevillea robusta* [30]
5	1322	(*Z*)-3-Hexenyl acetate	C_8_H_14_O_2_	82/67/54	3681-71-8	0.23	0.41	0.27	0.21	0.17	0.21	*Passiflora mollissima* [31]
6	1341	(*Z*)-2-Hexenyl acetate	C_8_H_14_O_2_	142/100/67/55	56922-75-9	0.41	0.69	0.40	0.43	0.37	0.49	-
7	1359	1-Hexanol	C_6_H_14_O	101/84/69/56	111-27-3	0.41	0.54	0.61	0.56	0.47	0.59	-
8	1375	(*E*,*Z*)-2,6-Dimethylocta- 2,4,6-triene	C_10_H_16_	136/121/105/79	7216-56-0	0.13	0.11	0.05	0.07	0.09	0.14	*Pistacia atlantica* [32]
9	1389	(*Z*)-4-Hexen-1-ol	C_6_H_12_O	100/82/67/55	928-91-6	1.90	2.19	2.11	1.29	1.31	1.40	-
10	1412	2-Hexen-1-ol	C_6_H_12_O	100/82/57	928-95-0	1.06	1.13	1.03	0.81	0.75	0.86	Olive oil [28]
11	1443	(*E*)-2-Methyl-2-vinyl- (1-hydroxy-1- methylethyl)tetrahydrofuran	C_10_H_18_O_2_	111/94/68/59	34995-77-2	0.50	0.51	0.43	0.41	0.37	0.40	Pu-erh tea [33]
12	1473	2,2,6-Trimethyl-6-vinyldihydro- 2H-pyran-3(4H)-one	C_12_H_20_O_3_	168/110/82/68	33933-72-1	30.28	35.82	27.44	28.91	28.34	32.95	-
13	1480	(*E*)-2-Hexenyl butyrate	C_10_H_18_O_2_	170/155/71/55	53398-83-7	0.07	0.08	0.07	0.06	0.06	0.05	Longjing tea [34]
14	1484	8-Isopropyl-1,3-dimethyltricyclo [4.4.0.0(2,7)]dec-3-ene	C_15_H_24_	204/161/119/ 105/81/55	138874-68-7	0.07	0.07	0.05	0.07	0.10	0.10	*Artemisia ordosica* [35]
15	1493	(*Z*)-3-Hexenyl pentanoate	C_11_H_20_O_2_	103/82/67/55	35852-46-1	0.09	0.13	0.24	0.09	0.05	0.06	*Lysimachia paridiformis* [36]
16	1506	(*E*)-2-Hexenyl pentanoate	C_11_H_20_O_2_	184/169/85/57	56922-74-8	0.19	0.22	0.41	0.20	0.16	0.16	-
17	1526	8,8-Dimethoxyoct-2-yl 2- formyl-4,6-dimethoxybenzoate	C_20_H_30_O_7_	209/193/165/71	312305-58-1	0.18	0.18	0.25	0.20	0.23	0.18	-
18	1556	3,7-Dimethyl-1,6-octadien-3-ol	C_10_H_18_O	136/121/ 93/71/55	78-70-6	0.03	0.02	0.02	0.02	0.01	0.01	La Rioja grape [37]
19	1588	(*E*)-4,11,11-Trimethyl- 8-methylene-bicyclo [7.2.0]undec-4-ene	C_15_H_24_	205/189/161/ 133/107/93/69	87-44-5	1.79	1.92	1.73	2.10	2.52	2.49	*Aquilegia japonica* [38]
20	1630	3-Acetoxy-2,2,6-trimethyl- 6-vinyltetrahydropyran	C_12_H_20_O_3_	197/179/155/ 137/94/68/55	67674-42-4	0.38	0.73	0.34	0.53	0.36	0.58	-
21	1660	1,5,9,9-Tetramethyl-1,4,7- cycloundecatriene	C_15_H_24_	204/189/161/ 147/121/93/80	515812-15-4	1.10	1.15	0.98	1.23	1.54	1.49	*Artemisia dracunculus* [39]
22	1670	(*Z*)-3-Hexenyl tiglate	C_11_H_18_O_2_	101/83/67/55	67883-79-8	0.08	0.10	0.04	0.16	0.29	0.29	*Gardenia jasminoides* [40]
23	1675	(*E*)-2-Hexenyl hexanoate	C_12_H_22_O_2_	198/169/99/71	53398-86-0	0.13	0.13	0.14	0.08	0.06	0.06	Longjing tea [34]
24	1733	Benzyl acetate	C_9_H_10_O_2_	150/128/91/79	140-11-4	0.26	0.16	0.28	0.24	0.21	0.20	*Prunus mume* [41]
25	1753	1-Isopropyl-7-methyl-4- methylene-1,2,3,4,4a,5,6,8a- octahydronaphthalene	C_15_H_24_	204/161/105	24268-39-1	0.45	0.45	0.35	0.45	0.56	0.60	*Baccharis tridentata* [42]
26	1775	2,2,6-Trimethyl-6- vinyltetrahydro-2H-pyran-3-ol	C_10_H_18_O_2_	170/152/137/ 109/94/68	14049-11-7	42.71	38.71	41.71	42.64	40.59	39.62	Pu-erh tea [33]
27	1783	Methyl N-hydroxy benzenecarboximidate	C_8_H_9_NO_2_	151/133/105/73	67160-14-9	0.45	0.36	0.91	0.58	0.48	0.38	Endophytic fungi from *Baliospermum montanum* [43]
28	1797	1-(2-Butoxyethoxy)ethanol	C_8_H_18_O_3_	132/100/75/57	54446-78-5	0.09	0.07	0.07	0.07	0.05	0.05	-
29	1812	(3*E*,7*E*)-4,8,12-Trimethyltrideca- 1,3,7,11-tetraene	C_16_H_26_	218/203/175/ 137/95/69/53	62235-06-7	0.35	0.34	0.34	0.23	0.16	0.23	*Invasive alligatorweed* [44]
30	1880	Phenylmethanol	C_7_H_8_O	108/91/79/65	100-51-6	1.33	0.68	1.36	0.94	1.06	0.74	*Prunus mume* [41]
31	1913	2-Phenylethanol	C_8_H_10_O	122/91/77/65	60-12-8	2.81	2.18	4.87	2.33	2.83	1.64	*Populus trichocarpa* [45]
32	1931	7,11,15-Trimethyl-3- methylenehexadec-1-ene	C_20_H_38_	278/263/137/ 123/95/68/57	504-96-1	0.17	0.11	0.40	0.28	0.33	0.25	*Herpetospermum pedunculosum* [13]
33	1949	Benzothiazole	C_7_H_5_NS	135/108/95/69	95-16-9	0.25	0.23	0.26	0.24	0.29	0.24	Tomato [27]
34	2022	1,3,3-Trimethyl-2- oxabicyclo[2.2.2]octan-6-ol	C_10_H_18_O_2_	170/143/126/ 108/93/71	60761-00-4	0.24	0.09	0.19	0.23	0.14	0.17	*Pleurotus ostreatus* and *Favolus tenuiculus* [46]
35	2026	3,7-Dimethylocta- 1,6-dien-3-yl formate	C_11_H_18_O_2_	182/165/136/ 121/93/69	115-99-1	0.12	0.09	0.13	0.10	0.06	0.06	-
36	2048	Isopentyl 2-hydroxybenzoate	C_12_H_16_O_3_	208/193/165/ 138/120/92	87-20-7	0.20	0.23	0.39	0.27	0.11	0.15	-
37	2053	(*E*)-3,7,11-Trimethyldodeca- 1,6,10-trien-3-ol	C_15_H_26_O	189/161/107/ 93/69	40716-66-3	0.14	0.10	0.15	0.14	0.13	0.11	*Echinacea* flower [47]
38	2078	Hexyl benzoate	C_13_H_18_O_2_	206/123/105/77	6789-88-4	0.60	0.58	0.71	0.67	0.44	0.42	*Salvia reuterana* [48]
39	2128	(*Z*)-3-Hexenyl benzoate	C_13_H_16_O_2_	105/82/67	25152-85-6	1.28	1.03	1.48	0.82	0.58	0.52	Apple [49]
40	2131	6,10,14-Trimethyl pentadecan-2-one	C_18_H_36_O	250/165/137/ 109/95/71/58	502-69-2	0.30	0.26	0.17	0.26	0.22	0.53	-
41	2141	4-(3,3-Dimethyloxiran-2-yl)-2- (oxiran-2-yl)butan-2-ol	C_10_H_18_O_3_	143/102/ 84/69/55	1365-19-1	0.15	0.05	0.11	0.12	0.06	0.10	Lemon grass oil [50]
42	2151	(*E*)-2-Hexenyl benzoate	C_13_H_16_O_2_	204/105/77	76841-70-8	0.64	0.32	0.56	0.45	0.43	0.33	-
43	2173	1,6-Dimethyl-4-propan-2-yl- 3,4,4a,7,8,8a-hexahydro- 2H-naphthalen-1-ol	C_15_H_26_O	204/189/161/ 147/119/105	5937-11-1	0.20	0.14	0.20	0.27	0.36	0.35	-
44	2213	Hexyl 2-hydroxybenzoate	C_13_H_18_O_3_	222/138/120/92	259-76-3	0.09	0.08	0.11	0.19	0.11	0.12	-
45	2236	4-Isopropyl-1,6-dimethyl- 1,2,3,4,4a,7,8,8a- octahydronaphthalen-1-ol	C_15_H_26_O	204/161/121/95	81-34-5	0.05	0.02	0.06	0.08	0.10	0.11	*Schisandra chinensis* [51]
46	2288	(*Z*)-Hex-3-en-1-yl 2- hydroxybenzoate	C_13_H_16_O_3_	220/138/120/ 82/67/55	65405-77-8	1.06	0.69	1.00	0.61	0.42	0.37	*Ulmus pumila* [52]
47	2296	2,3-Dihydroxypropyl acetate	C_5_H_10_O_4_	134/103/74	106-61-6	0.06	0.11	0.03	0.06	0.13	0.06	-
48	2349	4,4,7a-Trimethyl-5,6,7,7a- tetrahydrobenzofuran-2(4H)-one	C_11_H_16_O_2_	180/137/111/67	15356-74-8	0.06	0.05	0.07	0.09	0.06	0.07	-
49	2384	1-Methyl-4-[(2*Z*)-6- methylhepta-2,5-dien-2-yl]-7- oxabicyclo[4.1.0]heptane	C_15_H_24_O	107/93/79/55	121467-35-4	0.01	0.01	0.01	0.01	0.01	0.01	-
50	2446	Benzoic acid	C_7_H_6_O_2_	122/105/77/51	65-85-0	0.06	0.06	0.07	0.06	0.06	0.05	*Telfairia occidentalis* [53]
51	2556	Diisobutyl phthalate	C_16_H_22_O_4_	281/167/149/57	84-69-5	0.09	0.11	0.09	0.09	0.11	0.10	-
52	2743	Di(phenethyl) diglycolate	C_20_H_22_O_5_	342/104/77	84-69-5	0.79	0.33	1.50	0.98	1.75	0.56	-
53	2817	Benzyl 2-hydroxybenzoate	C_14_H_12_O_3_	228/109/91/65	118-58-1	0.71	0.25	0.54	1.39	1.38	0.66	-

RI: retention index; MF: molecular formula.

**Table 2:** The relative content of different types of volatile compounds in female and male *T. anguina* buds.

Type of Compounds	Number of Compounds	Relative Content/%								*p*-Value
		f1	f2	f3	$\bar{f}$	m1	m2	m3	$\bar{m}$	
Alcohols	8	9.63	9.48	12.36	10.49	8.60	8.89	7.59	8.36	0.10
Ketones	1	0.30	0.26	0.17	0.24	0.26	0.22	0.53	0.34	0.42
Non-aromatic esters	9	1.38	1.96	1.73	1.69	1.39	1.35	1.44	1.39	0.16
Aromatic esters	11	5.90	3.96	6.91	5.59	5.91	5.77	3.61	5.10	0.69
Monoterpenes	4	0.95	0.7	0.8	0.82	0.85	0.63	0.74	0.74	0.47
Sesquiterpenes	7	2.71	2.71	2.55	2.66	3.12	3.78	3.77	3.56	0.02
Diterpenes	1	0.17	0.11	0.4	0.23	0.28	0.33	0.25	0.29	0.55
Alkenes	5	2.37	2.4	1.99	2.25	3.86	6.55	5.62	5.34	0.02
Oximes	1	0.45	0.36	0.91	0.57	0.58	0.48	0.38	0.48	0.63
Heterocycles	4	73.62	75.49	69.75	72.95	72.32	69.58	73.39	71.76	0.59
Alkanes	1	0.23	0.30	0.32	0.28	0.21	0.19	0.23	0.21	0.07
Acids	1	0.06	0.06	0.07	0.06	0.06	0.06	0.05	0.06	0.23

**Table 3:** Differential volatile compounds between female and male buds of *T. anguina*.

Section	No.	Compound	VIP
A	4	(*E*)-4,8-Dimethyl-1,3,7-nonatriene	3.68
A	19	(*E*)-Caryophyllene	1.57
A	21	1,5,9,9-Tetramethyl-1,4,7-cycloundecatriene	1.25
B	9	(*Z*)-4-Hexen-1-ol	1.81
B	39	(*Z*)-3-Hexenyl benzoate	1.60
B	46	(*Z*)-3-Hexenyl salicylate	1.30
B	10	2-Hexen-1-ol	1.10

A: higher in males than in females; B: higher in females than in males.

## Data Availability

All the relevant data have been provided in the manuscript. The authors will provide additional details if required.
